# Supplementary material for: An allosteric ligand-binding site in the extracellular cap of K2P channels
Source: Nat Commun. 2017 Aug 29;8:378. doi: 10.1038/s41467-017-00499-3 (PMC5575254; doi:10.1038/s41467-017-00499-3)
Supplement: Supplementary file 1 — Supplementary Information [file 41467_2017_499_MOESM1_ESM.pdf]

File Name: Supplementary Information
Description: Supplementary Figures and Supplementary Tables

File Name: Peer Review File
Description:

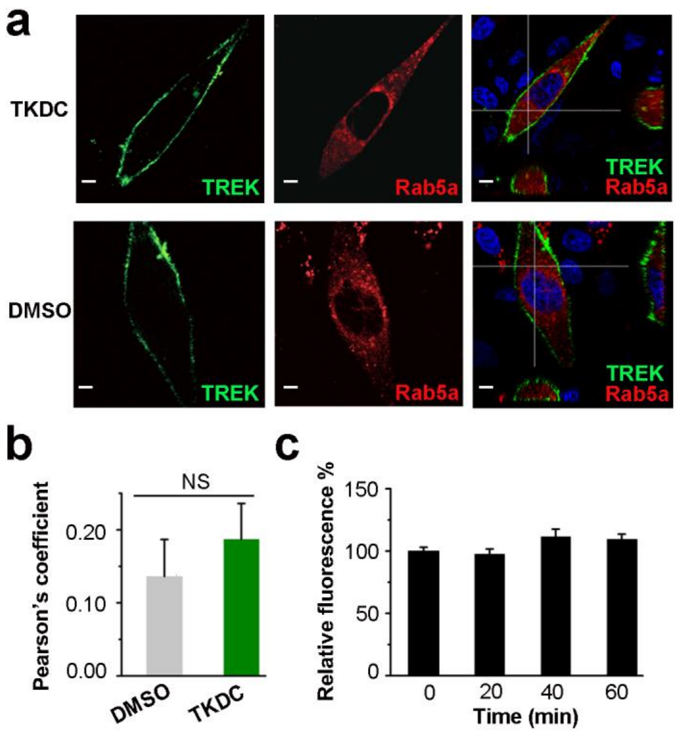

Supplementary Figure 1 | TREK-1 internalization from cell surface

(**a**) Internalization of TREK-1 from cell surface induced by TKDC. CHO-K1 cells transiently expressing TREK-1 and orange fluorescent protein (OFP)-tagged Rab5a were incubated with 10 µM TKDC and DMSO as control. Nuclei were counterstained with 4,6-diamidino-2-phenylindole (DAPI). 3D reconstruction was done by Imaris software. White scale bar represents 10 µm. Co-localization between TREK1-GFP and OFP-tagged Rab5a was analyzed using Imaris software. (**b**) Pearson's correlation coefficient calculated from 18 fields. The unpaired t-test was used for statistical analysis [t (26) = 0.68 and n = 18]. (**c**) Flow cytometry analysis of TREK-1 internalization. The relative fluorescence represented the relative mean fluorescence intensity from three independent experiments each done in duplicate. The paired t-test was used for statistical analysis [t (3) = 0.69 and n = 6 for 20 min, t (3) = 0.29 and n = 6 for 40 min, t (3) = 0.23 and n = 6 for 60 min]. The data are shown as the mean ± s.e.m.

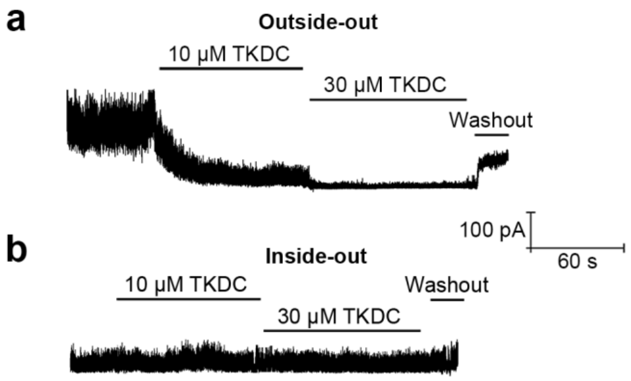

Supplementary Figure 2 | TKDC-induced inhibition of TREK-1(**a**) Inhibitory effects of 10 μM and 30 μM TKDC on TREK-1 in outside-out configuration. (**b**) Inhibitory effects of 10 μM and 30 μM TKDC on TREK-1 in inside-out configuration. Traces were obtained with pipette voltage of +80 mV. The bold bar above the current trace shows the application and washout of TKDC.

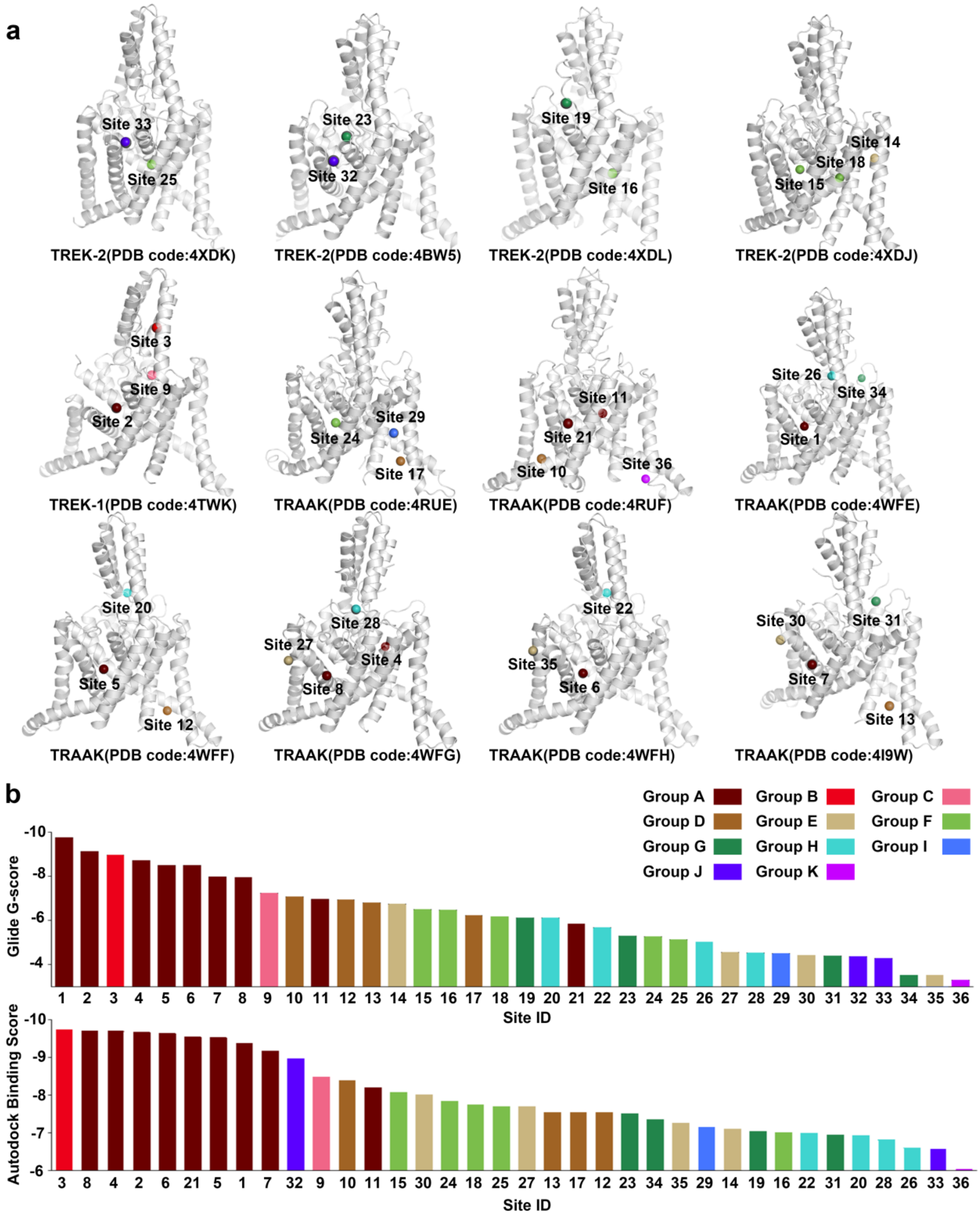

Supplementary Figure 3 | Docking of TKDC to TREK channels

(**a**) Potential ligand-binding sites in the crystal structures of TREK channels. Each predicted binding site is indicated as a sphere. (**b**) Glide G-scores and Autodock binding scores of docking TKDC to each potential binding site. The binding sites were assigned in the different groups according to their locations and compositions of residues. Eleven different groups are shown in different colors.

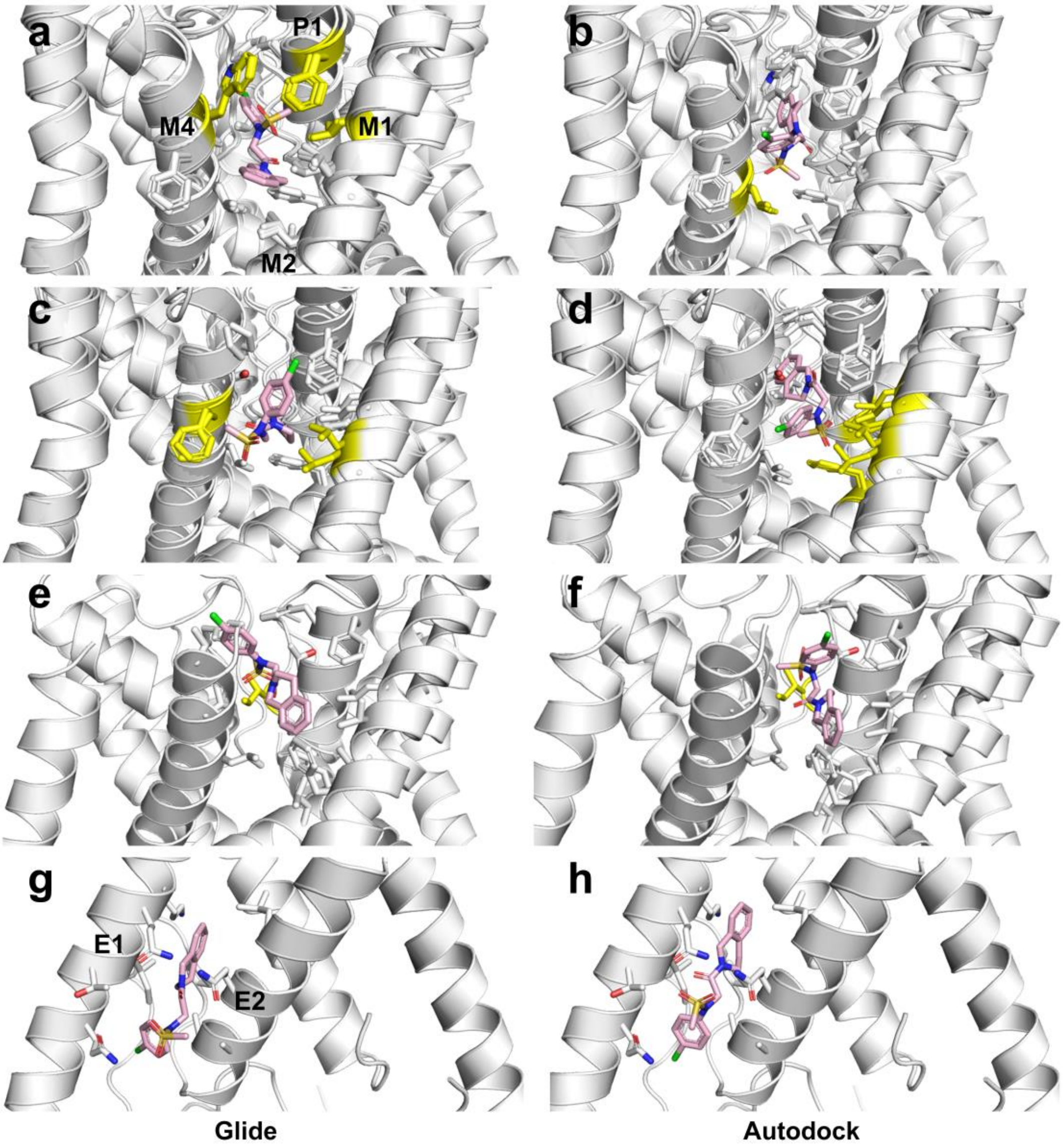

Supplementary Figure 4 | Docking models of TKDC in the binding sites of groups A and B

(**a-f**) Representative docking poses of TKDC in the group A sites, including (**a, b**) sites 1, 4, 5, 6, 7 and 8, (**c, d**) sites 11 and 21, and (**e, f**) site 2. (**g, h**) Representative docking poses of TKDC in the group B site 3. These models were generated using (**a, c, e, g**) Glide and (**b, d, f, h**) Autodock. TKDC and protein residues in the binding sites are shown as sticks. The hydrophobic residues interacting with the charged sulfonyl group of TKDC are highlight in yellow. Residues blocking view are omitted.

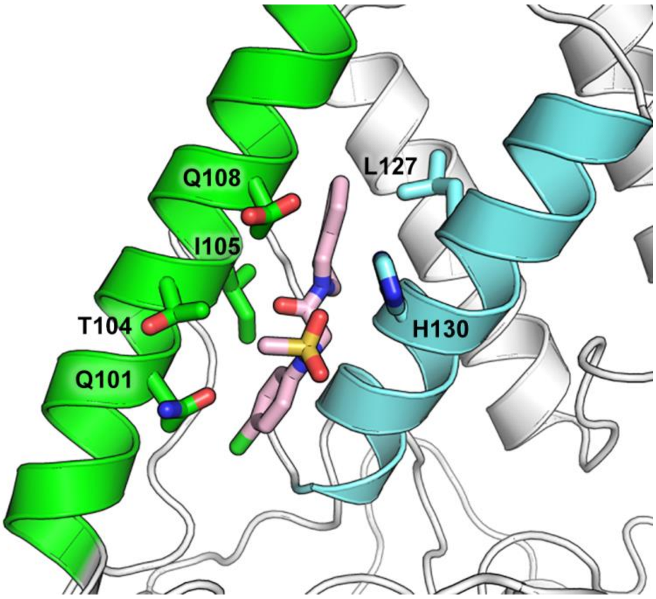

Supplementary Figure 5 | Binding mode of TKDC to TREK-2

TKDC and protein residues in the extracellular binding site are shown as sticks. The E1 and E2 helices are shown as green and cyan cartoons. RosettaLigand was applied to docking.

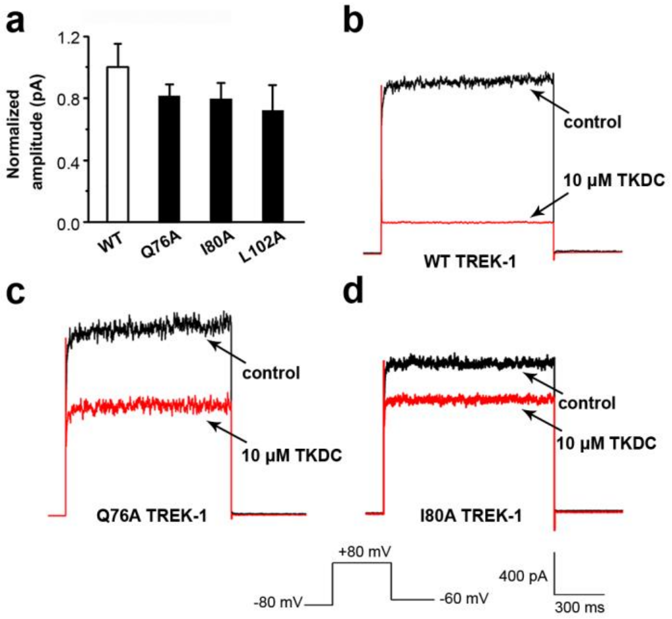

Supplementary Figure 6 | Inhibitory effects of TKDC on the mutant TREK-1 channels

(**a**) Normalized current amplitudes of the WT and mutant TREK-1 channels in the control group without the effect of TKDC. The data are shown as the mean ± s.e.m. (**b-d**) Representative traces of currents recorded from CHO cells transfected with WT (**b**) and mutant TREK-1 channels (**c, d**).

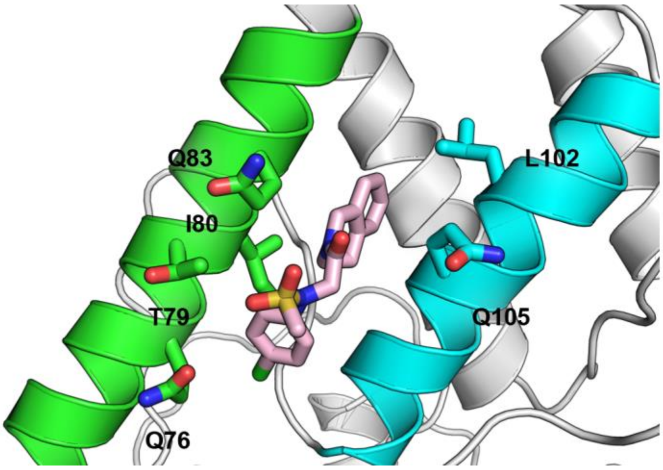

Supplementary Figure 7 | Binding mode of TKDC to TREK-1 in MD simulations

Representative simulation model of TREK-1/ TKDC complex from the last 200- ns MD simulations of the complex system $S_{complex}$. TKDC and protein residues in the binding site are shown as sticks. The E1 and E2 helices are shown as green and cyan cartoons.

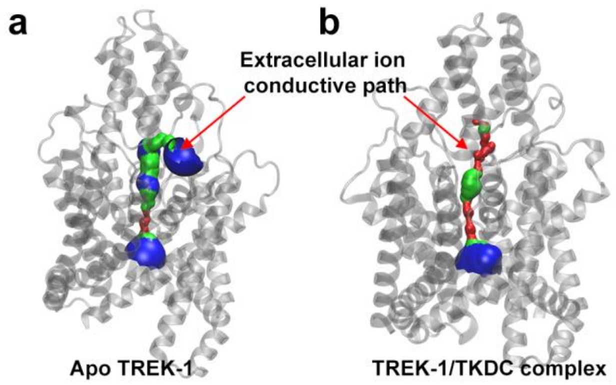

Supplementary Figure 8 | Conductive pathway of TREK-1 in simulation systems

(**a**) Conformation of TREK-1 in the apo system S_{apo}. (**b**) Conformation of TREK-1 in the complex system $S_{complex}$ with TKDC. The protein is shown as a grey cartoon. HOLE color code is used to present ion conductive pathway: blue, radius > 1.2 Å; green, radius > 0.6-1.2 Å; red, radius ≤ 0.6 Å. All illustrations are from the end of simulation runs.

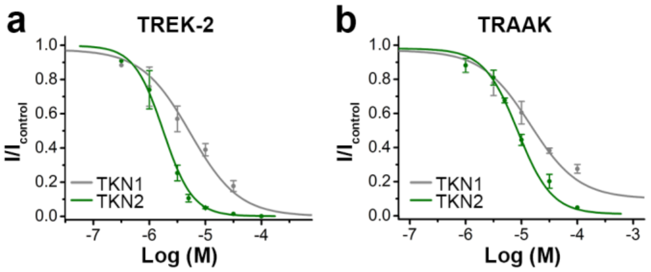

Supplementary Figure 9 | Inhibition of TREK-2 and TRAAK by TKN1 and TKN2.

(**a**) Dose-dependent inhibition of TREK-2 by TKN1 and TKN2. (**b**) Dose-dependent inhibition of TRAAK by TKN1 and TKN2. IC_{50} values were obtained by dose-response fitting.

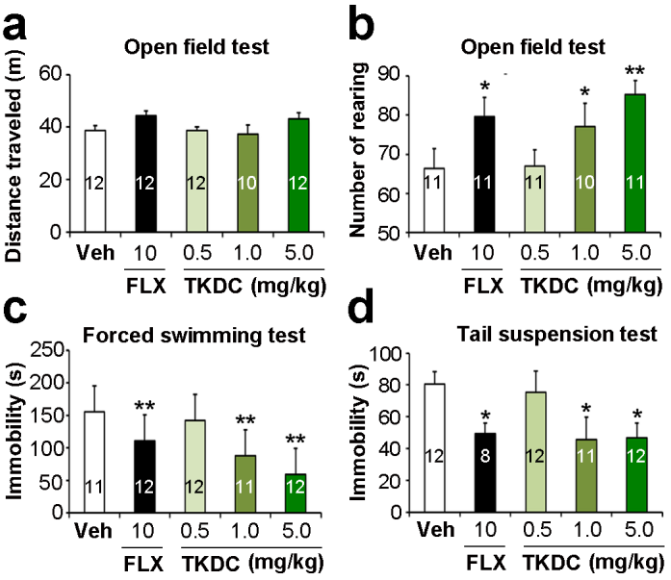

Supplementary Figure 10 | Acute administration of TKDC in mice

(**a**) The statistical analysis of total distances traveled after drugs administration reflection of the locomotion activity in the open field test [one-way ANOVA with post hoc LSD test, F (4, 50) = 1.58]. Veh represented the vehicle. FLX represented the fluoxetine administered at a dose of 10 mg kg^{-1}. TKDC was administered at a dose of 0.5 mg kg^{-1}, 1 mg kg^{-1} and 5 mg kg^{-1}, respectively. (**b**) Histogram indicated the number of rearing behaviors in the open field test [one-way ANOVA with post hoc LSD test, F (4, 53) = 3.15]. (**c**) Histogram indicated the duration of immobility in the forced swimming test [one-way ANOVA with post hoc LSD test, F (4, 53) = 8.90]. (**d**) Histogram indicated the duration of immobility in the tail suspension test [one-way ANOVA with post hoc LSD test, F (4, 49) = 2.71]. The numbers in the bars indicate the number of cells studied per condition. The results are shown as the mean ± s.e.m; * indicates $P < 0.05$, ** indicates $P < 0.01$.

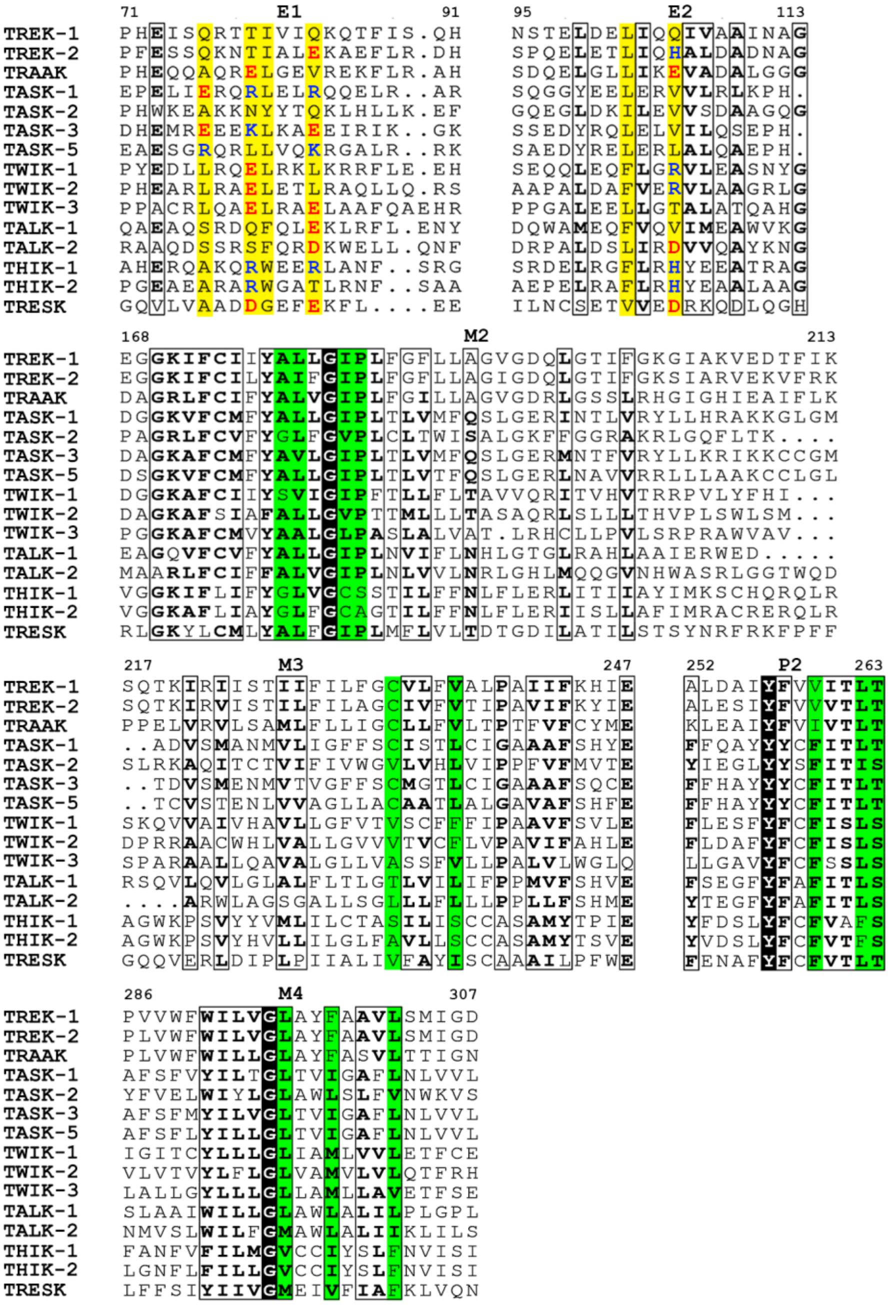

Supplementary Figure 11 | Multiple sequence alignments of 15 human K2P channels. Residues in E1-E2, M2-M4 and P2 helices are included. Residues in TKDC-binding site are highlight in yellow color. Residues in fluoxetine-binding site are highlight in green color. In the TKDC-binding site, negatively charged residues are colored in red, positively charged residues are in blue. Conserved residues are marked with boxes.

Supplementary Table 1 | Inhibitory effects of TKDC on the mutant TREK-1 channels

Construct	IC_{50} (μM)	Number of recorded cells
T79A TREK-1	2.4 ± 0.4	6
Q83A TREK-1	2.4 ± 0.6	4
Q105A TREK-1	2.2 ± 0.5	4

Supplementary Table 2 | Average minimal distance between the bottom of E2 helix and E1 helix

Simulation ID	Distance (nm)*
S_{apo} system simulation 1	0.71±0.04
S_{apo} system simulation 2	0.73±0.05
$S_{complex}$ system simulation 1	0.96±0.03
$S_{complex}$ system simulation 2	0.82±0.05
$S_{complex}$ system simulation 3	0.87±0.05

* indicates only backbone heavy atoms and the last 200-ns trajectories of simulations were accounted in the calculation.

Supplementary Table 3 | Inhibition of TREK-2 and TRAAK channels by TKN1 and TKN2.

Construct	Inhibitor	IC_{50} (μM)	Number of recorded cells
TREK-2	TKN1	4.4 ± 1.0	6
TREK-2	TKN2	1.7 ± 0.8	3
TRAAK	TKN1	15.7 ± 3.1	6
TRAAK	TKN2	10.3 ± 1.5	6

Supplementary Table 4 | List of primers used in the study.

**Construct**	**Primer ID**	**Sequence (5’ > 3’)**
Q76A	F-Q76A	TCATGAGATTTCAGCGAGGACCACCATTGTGATCCAG
TREK-1-pEGFPN1	R-Q76A	ATGGTGGTCCTCGCTGAAATCTCATGAGGCTGCTCC
T79A	F-T79A	GGAGCAGCCTCATGAGATTTCACAGAGGACCACCATTG
TREK-1-pEGFPN1		TGATCCAGAAGCAAACATTCATATC
	R-T79A	GATATGAATGTTTGCTTCTGGATCACAATGGTGGTCCTC
		TGTGAAATCTCATGAGGCTGCTCC
I80A	F-I80A	GAGGACCACCGCTGTGATCCAGAAGCAAACATTCAT
TREK-1-pEGFPN1	R-I80A	CTTCTGGATCACAGCGGTGGTCCTCTGTGAAATCTC
Q83A	F-Q83A	ACCATTGTGATCGCGAAGCAAACATTCATATCCCAACAT
TREK-1-pEGFPN1	R-Q83A	TGAATGTTTGCTTCGCGATCACAATGGTGGTCCTCTG
L102A	F-L102A	GAGCTGGATGAAGCCATTCAGCAAATAGTGGCAGCAAT
TREK-1-pEGFPN1	R-L102A	CTATTTGCTGAATGGCTTCATCCAGCTCCGTCGAATTG
Q105A	F-Q105A	GAACTCATTCAGGCAATAGTGGCAGCAATAAATGCAGG
TREK-1-pEGFPN1	R-Q105A	GCTGCCACTATTGCCTGAATGAGTTCATCCAGCTCC
A35Q	F-A35Q	GCCCCACGAGCAGCAGCAGCAGAGGGAGCTGGGGGAG
TRAAK-pEGFPN1		GTCCGAGAGAAGT
	R-A35Q	CCTCAGGAACTTCTCTCGGACCTCCCCCAGCTCCCTCT
		GCTGCTGCTGCTCGTGGGGCTGCT
E38T	F-E38T	CACGAGCAGCAGGCCCAGAGGACCCTGGGGGAGGTCC
TRAAK-pEGFPN1		GAGAGAAGT
	R-E38T	CAGGAACTTCTCTCGGACCTCCCCCAGGGTCCTCTGGG
		CCTGCTGCTCGTG
V42Q	F-V42Q	GCCCAGAGGGAGCTGGGGGAGCAGCGAGAGAAGTTCC
TRAAK-pEGFPN1		TGAGGGCCCATC
	R-V42Q	CTCAGGAACTTCTCTCGCTGCTCCCCCAGCTCCCTCTGG
		GCCT
